# Oncogenic activation of *PIK3CA* in cancers: Emerging targeted therapies in precision oncology

**DOI:** 10.1016/j.gendis.2024.101430

**Published:** 2024-09-10

**Authors:** Yuxiang Wang, Valery Rozen, Yiqing Zhao, Zhenghe Wang

**Affiliations:** a Department of Genetics and Genome Sciences, Case Western Reserve University, Cleveland, OH 44106, USA; b Case Comprehensive Cancer Center, Case Western Reserve University, Cleveland, OH 44106, USA; c College of Human Medicine, Michigan State University, Grand Rapids, MI 49503, USA

**Keywords:** Hotspot mutation, Isoform/mutant-specific inhibitors, Metabolism, *PIK3CA*, Tumor microenvironment

## Abstract

Phosphoinositide 3-kinases (PI3Ks) are heterodimers consisting of a p110 catalytic subunit and a p85 regulatory subunit. The *PIK3CA* gene, which encodes the p110α, is the most frequently mutated oncogene in cancer. Oncogenic *PIK3CA* mutations activate the PI3K pathway, promote tumor initiation and development, and mediate resistance to anti-tumor treatments, making the mutant p110α an excellent target for cancer therapy. *PIK3CA* mutations occur in two hotspot regions: one in the helical domain and the other in the kinase domain. The *PIK3CA* helical and kinase domain mutations exert their oncogenic function through distinct mechanisms. For example, helical domain mutations of p110α gained direct interaction with insulin receptor substrate 1 (IRS-1) to activate the downstream signaling pathways. Moreover, p85β proteins disassociate from helical domain mutant p110α, translocate into the nucleus, and stabilize enhancer of zeste homolog 1/2 (EZH1/2). Due to the fundamental role of PI3Kα in tumor initiation and development, PI3Kα-specific inhibitors, represented by FDA-approved alpelisib, have developed rapidly in recent decades. However, side effects, including on-target side effects such as hyperglycemia, restrict the maximum dose and thus clinical efficacy of alpelisib. Therefore, developing p110α mutant-specific inhibitors to circumvent on-target side effects becomes a new direction for targeting *PIK3CA* mutant cancers. In this review, we briefly introduce the function of the PI3K pathway and discuss how *PIK3CA* mutations rewire cell signaling, metabolism, and tumor microenvironment, as well as therapeutic strategies under development to treat patients with tumors harboring a *PIK3CA* mutation.

## Introduction

Phosphoinositide 3-kinases (PI3Ks) are mediators of signaling cascades involved in the regulation of multi-process signal transductions implicated in cellular physiological functions, including cell proliferation, survival, metabolism, and migration.^1^ Class I PI3Ks are heterodimers consisting of a p110 catalytic subunit and a p85 regulatory subunit. *PIK3CA*, which encodes the p110α catalytic subunit, was first discovered to be frequently mutated in a variety of human cancers by a systematic sequencing of all 16 members of lipid kinases.^2^ This groundbreaking discovery laid the foundation for the FDA approval of the p110α-specific inhibitor alpelisib to treat *PIK3CA*-mutated, hormone receptor-positive (HR^+^), epidermal growth factor receptor 2-negative (HER2^−^) breast cancer patients, in combination with fulvestrant, an antagonist of the estrogen receptor (ER).^3^ Here, we discuss how *PIK3CA* mutations rewire cell signaling, metabolism, and tumor microenvironment, as well as therapeutic strategies that are under development to treat patients with tumors harboring a *PIK3CA* mutation.

## PI3K signaling pathways

**Figure 1:**
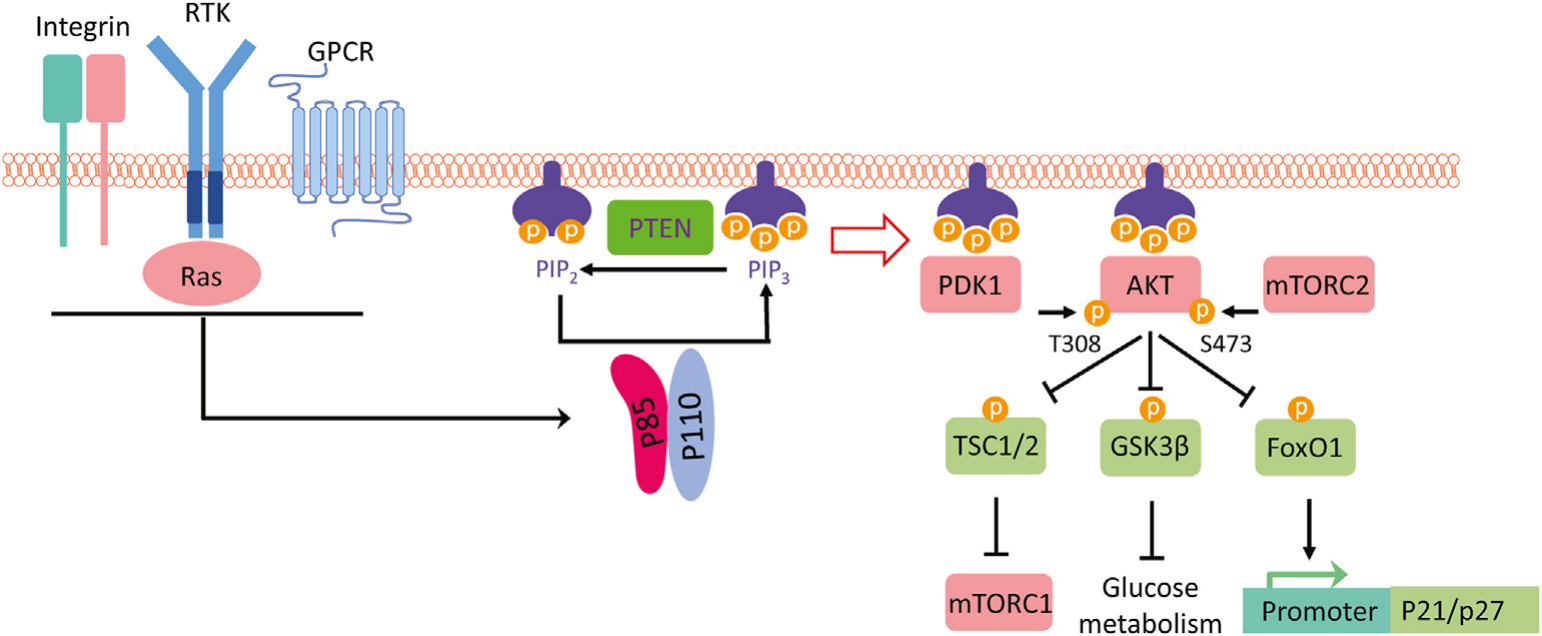
PI3K signaling pathways. Upon activation by RTK, GPCR, Integrin, and Ras, p110 loses inhibition by p85 and converts PIP2 to PIP3. PIP3 recruits AKT to the cell membrane, where AKT is activated through phosphorylation by PDK1 at T308 and by mTORC2 at S473. Activated AKT phosphorylates TSC1/2 and relieves its inhibition function on mTORC1, promoting protein translation. AKT also phosphorylates and inhibits GSK3β, initiating glucose metabolism and glycogen synthesis. AKT regulates the cell cycle by phosphorylating FoxO1 and decreasing its transcription of CDK inhibitors p21 and p27. AKT, protein kinase B; CDK, cyclin dependent kinase; FoxO1, forkhead box O1; GPCR, G-protein coupled receptor; GSK3β, glycogen synthase kinase 3β; mTORC1/2, mammalian target of rapamycin complex 1/2; PDK1, phosphoinositide-dependent kinase 1; PI3K, phosphoinositide 3-kinase; PIP2, phosphatidylinositol (4,5)-bisphosphate; PIP3, phosphatidylinositol (3,4,5)-trisphosphate; RTK, receptor tyrosine kinase; TSC1/2, tuberous sclerosis complex 1/2.

Insulin receptor (IR) and other receptor tyrosine kinases (RTKs) form dimers and lead to autophosphorylation at C-terminal domain tyrosine residues upon binding by their ligands.^4^ Activated RTKs then recruit PI3K to the plasma membrane through the Src homology 2 (SH2) domain of p85 directly or by phosphorylating specific adapter proteins such as the insulin receptor substrate 1 (IRS1) (Fig. 1). Upon binding to phosphorylated RTK or IRS1, p85 loses its inhibitory effector on p110, which phosphorylates phosphatidylinositol (4,5)-bisphosphate (PIP2) to produce the second messenger phosphatidylinositol (3,4,5)-trisphosphate (PIP3). PIP3 recruits serine/threonine protein kinase AKT (also known as protein kinase B, PKB) to the plasma membrane, where it is activated via phosphorylation of T308 by phosphoinositide-dependent kinase 1 (PDK1) and phosphorylation of S473 by mammalian target of rapamycin complex 2 (mTORC2). All three AKT isoforms (AKT1, AKT2, and AKT3) are activated by this same mechanistic pathway. AKT1 has a critical role in cancer metastasis and angiogenesis, AKT2 is involved in insulin metabolism, and AKT3 is mainly found in the brain cortex and hippocampus and is essential in regulating brain size. Phosphorylation of the three AKT isoforms leads to cell function influence regarding proliferation and metabolism.^5^ It is also vital to highlight three important substrates that interact highly with AKT: tuberous sclerosis complex 1/2 (TSC1/2), glycogen synthase kinase 3β (GSK3β), and forkhead box O1 (FoxO1).^6^ TSC1/2 plays a fundamental role in regulating PI3K signaling and inhibiting mTORC1 via GTPase activation, leading to essential information for protein processing and translation.^7^ GSK3β regulates glucose metabolism and is involved in crucial processes in many psychiatric and neurological diseases, mainly involved in proteasomal degradation.^8^ FoxO1 transcript factor acts as a tumor suppressor by promoting the expression of cyclin-dependent kinase inhibitors, p21Cip1 and p27Kip1.^9^ In the setting of cancer, loss of FoxO1 leads to increased cellular survival and tumorigenesis stimulation.^10^ In addition to RTKs, the PI3K pathway can also be activated by G-protein coupled receptors, integrins, cytokine/chemokine receptors, and B/T-cell receptors.^11^ Therefore, the PI3K pathway regulates cell proliferation, survival, metabolism, migration, and immune response.^1^ Moreover, class I PI3K catalytic isoforms have direct interactions with RAS superfamily proteins via RAS-binding domains.^12^ As a result, oncogenic RAS proteins have the ability to recruit PI3Kα to the cell membrane and stimulate increased activation of the p110α catalytic subunit.^13^ PI3K signal is mainly compromised by phosphatase and tensin homolog (PTEN), which catalyzes the conversion of PIP3 to PIP2.^14^

## *PIK3CA*/p110α helical domain mutation rewires cell signaling

**Figure 2:**
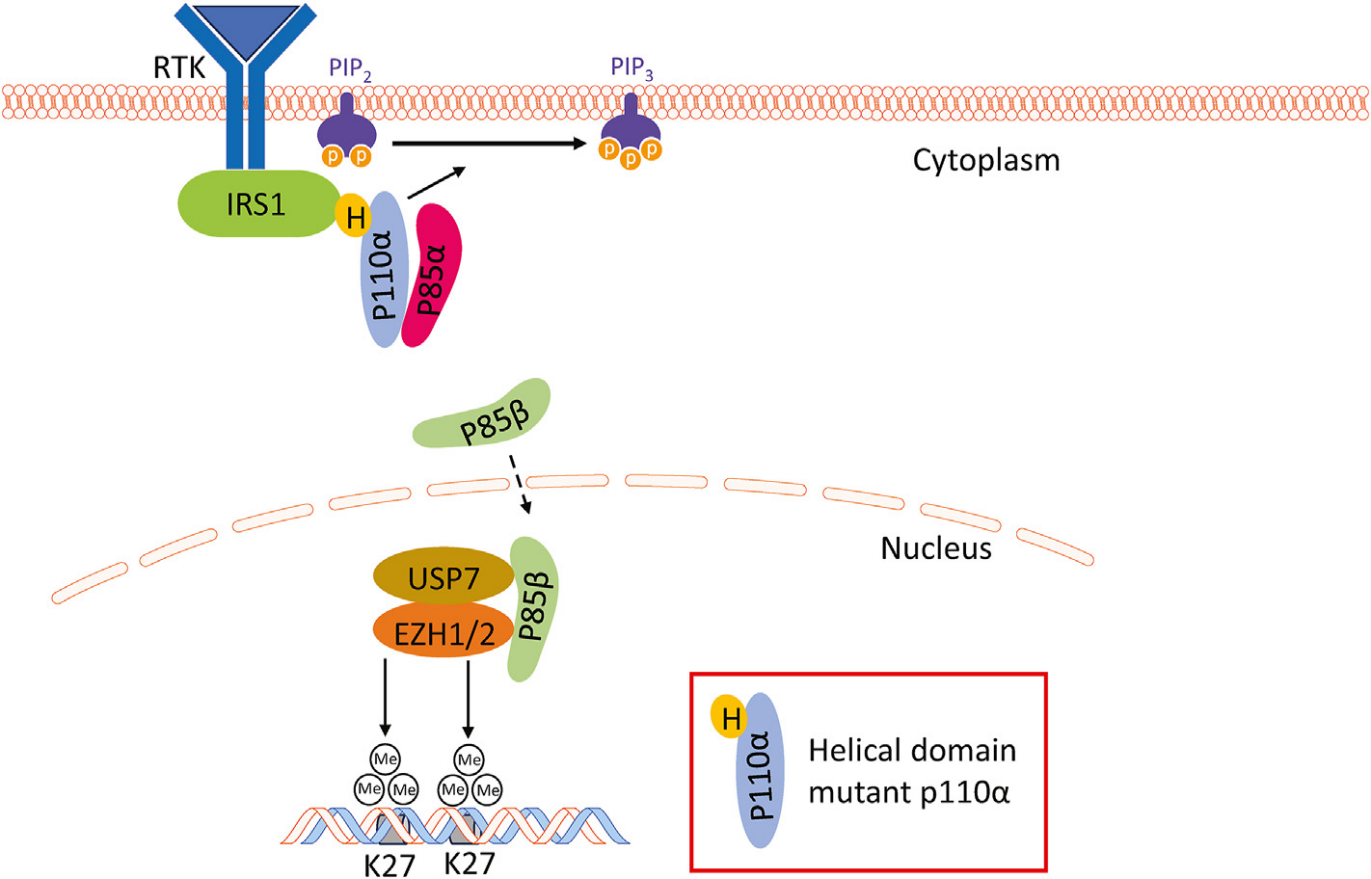
*PIK3CA* helical domain mutation rewires cell signaling. Helical domain mutation of p110α gains interaction with IRS1 and loses interaction with p85α/β. p110α with helical domain mutation is brought to cell membrane by IRS1 independent of p85, where it converts PIP2 to PIP3. The released p85β but not p85α translocates into the nucleus and stabilizes EZH1/2 by recruiting deubiquitylating enzyme USP7. EZH1/2, enhancer of zeste homolog 1/2; IRS1, insulin receptor substrate 1; PIK3CA, phosphatidylinositol-4,5-bisphosphate 3-kinase catalytic subunit alpha; PIP2, phosphatidylinositol (4,5)-bisphosphate; PIP3, phosphatidylinositol (3,4,5)-trisphosphate; RTK, receptor tyrosine kinase; USP7, ubiquitin-specific-processing protease 7.

PI3K signaling is one of the most frequently aberrantly activated pathways in cancer, mainly including activating mutation of *PIK3CA* and loss-of-function mutation of *PTEN*.^15^ Interestingly, the TCGA studies found that *PIK3CA* is the most frequently mutated kinase, with 17% of human cancers harboring a *PIK3CA* mutation.^16^ The p110α subunit of PI3Kα, coded by *PIK3CA* gene, is composed of five domains: an N-terminal adaptor-binding domain (ABD), a Ras-binding domain (RBD), a C2 domain, a helical domain, and a kinase domain.^17^ Most *PIK3CA* mutations occur in two hotspots: one in the helical domain (E542K, E545K, and Q546 K/P) and the other in the kinase domain (H1047R).^18^ Both helical domain and kinase domain hotspot mutations improve phosphorylated AKT levels and promote proliferation and resistance to apoptosis. The rare mutations in C2 domain (N345K, C420R, and E453K/Q) and ABD (R38 H/C, R88Q, R93Q, R108H, and G118/D) strengthen helical and kinase domain mutation through interrupting iSH2-C2/ABD interaction.^19^ In addition, helical domain and kinase domain mutations activated the PI3K signaling pathway synergically, indicating different mechanisms underlying helical domain and kinase domain mutations.^20^ To this end, we found two distinct mechanisms by which the helical domain mutated p110α rewires signaling pathways (Fig. 2). Firstly, the helical domain mutant p110α proteins, but not p110α kinase domain mutants, gain the ability to associate with IRS1 independent of the p85 regulatory subunit, thereby activating the downstream signaling pathways without growth factor stimulation.^21^ Disruption of the IRS1-p110α helical domain mutant protein–protein interaction by hydrocarbon-stapled p110α mutant peptides destabilized the p110α helical domain mutant protein, reduced AKT phosphorylation, and slowed xenograft tumor growth of a cancer cell line expressing a p110α helical domain mutation.^21^^,^^22^ Secondly, p85β, but not p85α, disassociates from p110α helical domain mutant proteins, translocated to the nuclear, and recruits deubiquitinase ubiquitin-specific-processing protease 7 (USP7) to stabilize EZH1/2^23^. Moreover, a combination of p110α and EZH2 inhibitors induces regression of tumors with *PIK3CA* helical domain mutations in multiple xenograft models,^23^ suggesting that this combination may be an effective approach to treat patients whose tumors harbor *PIK3CA* helical domain mutation. Consistently, a genome-wide gain-of-function clustered regularly interspaced short palindromic repeats (CRISPR)-CRISPR associated protein 9 (Cas9) screening also demonstrated that EZH2 mediated resistance to PI3Kα inhibitor CYH33 in KYSE510 cells harboring *PIK3CA*^E545K^ mutation.^24^

The kinase domain mutation H1047R has been reported to disrupt the conformation of the C-terminal tail of p110α, making it easier to switch from buried to disengaged conformation, according to the structures obtained through X-ray crystallography and cryoEM.^25^, ^26^, ^27^, ^28^ In addition, the H1047R mutation renders the C-terminal tail an additional positive charge and promotes its interactions with the membrane.^25^ Higher cell membrane affinity of p110α facilitates binding to its substrate PIP_2_ for catalysis.^19^ Active membrane-latched Ras also activates PI3Kα by enhancing its membrane interaction, which explains why helical domain mutation E542/5K depends on RAS-binding to enhance AKT phosphorylation and can be rescued by H1047R mutation when RBD mutated.^20^ Structurally, the helical domain mutation of p110 mimicked the function of phosphorylated tyrosine (pY) peptides, which releases autoinhibition by the nSH2 domain of p85.^19^ The mechanism is verified by the finding that while the H1047R mutant can be further activated in the presence of growth factor stimulation, the E545K mutant cannot be further activated.^29^

## *PIK3CA* mutations reprogram cancer metabolism

**Figure 3:**
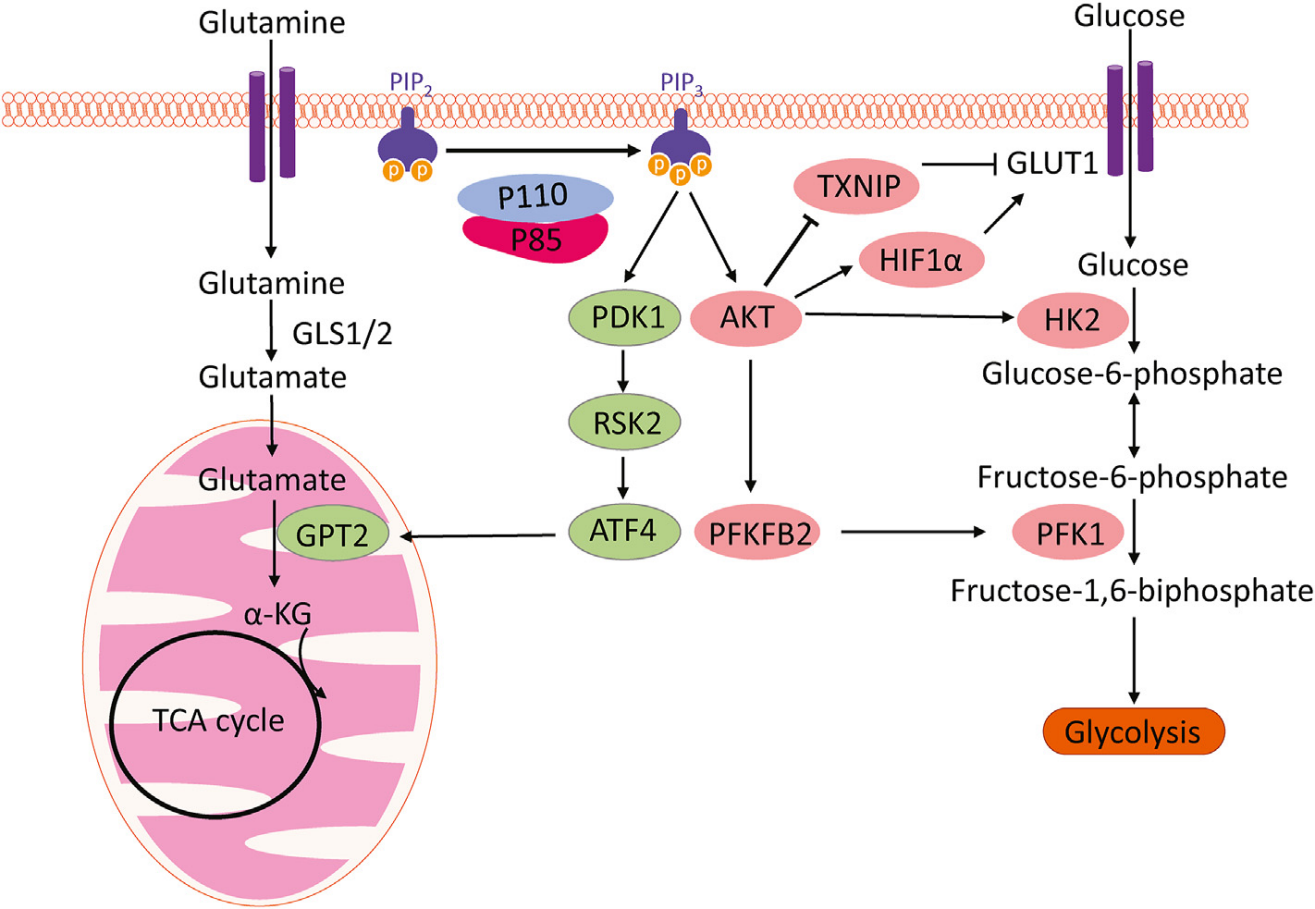
*PIK3CA* mutations reprogram cancer metabolism. PI3K activation renders cancer cells dependent on glutamine metabolism through the AKT-independent PDK1-RSK2-ATF4 axis. Transcript factor ATF4 increases the expression of GPT2, accelerating conversion from glutamate to α-KG in mitochondria. α-KG then goes into the tricarboxylic acid (TCA) cycle and fuels the cancer cell. PI3K activation evaluates the ability to use glucose as a fuel through multiple effectors. PI3K increases the pumping of glucose through the up-regulating expression of glucose transporter GLUT1 by HIF1α. Meanwhile, PI3K blocked endocytosis of GLUT1 by inhibiting TXNIP, maintaining GLUT1 on cell membranes. AKT activates HK2, which converts glucose to cell membrane impenetrable glucose-6-phosphate and accumulates glucose-6-phosphate in the cytoplasm for ATP production. AKT promotes another important reaction by activating PFKFB2 to produce a potent allosteric activator of PFK1. α-KG, alpha-ketoglutarate; AKT, protein kinase B; ATF4, activating transcription factor 4; GLS1, kidney-type glutaminase; GLS2, liver-type glutaminase; GLUT1, glucose transporter 1; GPT2, glutamate pyruvate transaminase 2; HIF1α, hypoxia-inducible factor 1α; HK2, hexokinase 2; PDK1, phosphoinositide-dependent kinase 1; PFK1, phosphofructokinase 1; PFKFB2, 6-phosphofructo-2-kinase/fructose-2,6-biphosphatase; PIK3CA, phosphatidylinositol-4,5-bisphosphate 3-kinase catalytic subunit alpha; PI3K, phosphoinositide 3-kinase; RSK2, ribosomal s6 kinase 2; TXNIP, thioredoxin-interacting protein.

**Figure 4:**
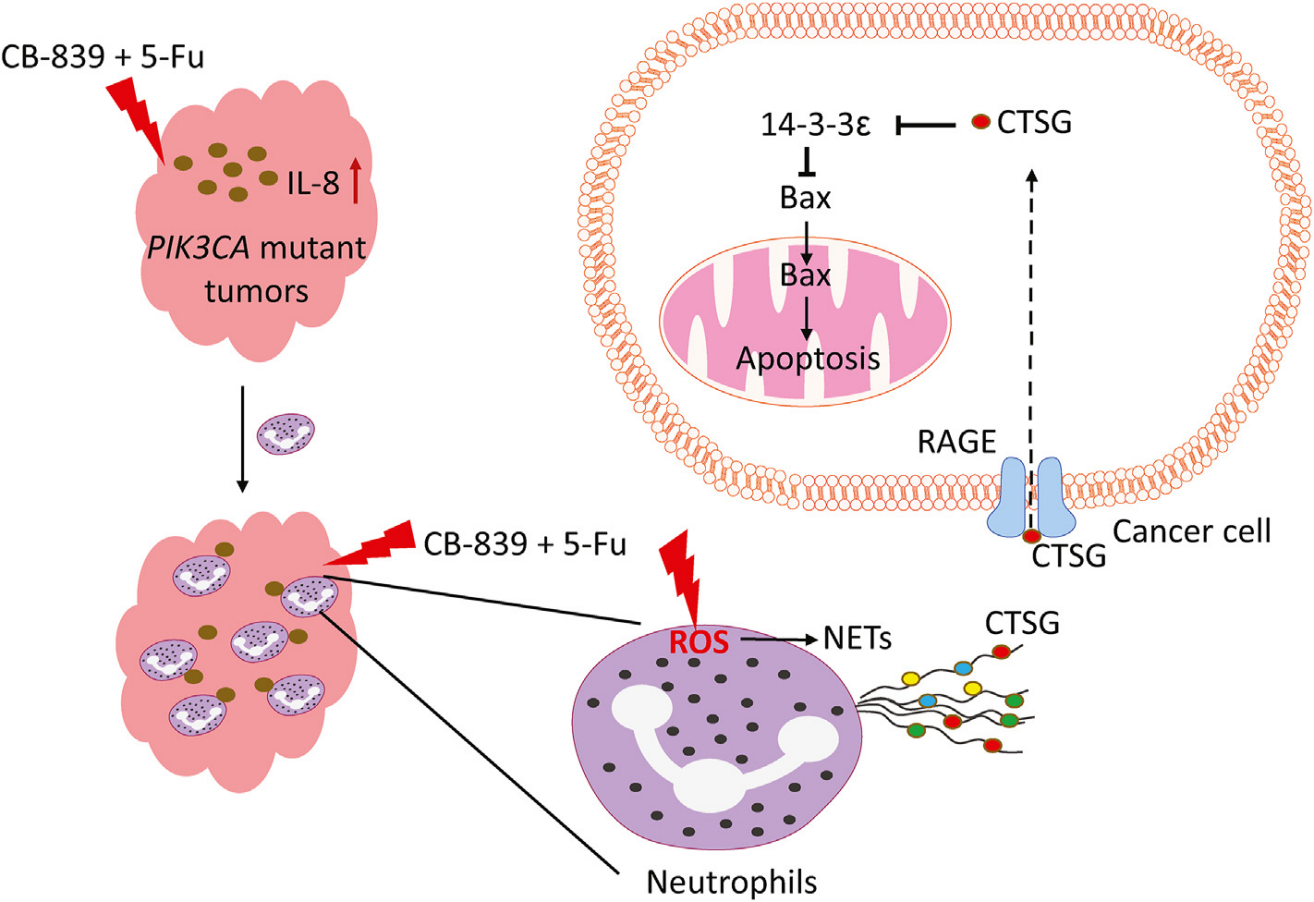
Combined CB-839 and 5-FU treatment induces NET release to kill cancer cells. Combined CB-839 and 5-FU treatment stimulates IL-8 secretion in *PIK3CA* mutant tumors but not in *PIK3CA* wild-type tumors. IL-8 then recruits neutrophils into tumors, where combined CB-839 and 5-FU treatment evaluates reactive oxygen species levels in neutrophils and promotes NET release. CTSG is released during NET formation and enters cancer cells in a RAGE-dependent manner. CTSG cleavages 14-3-3ε in cancer cells, releasing Bax to mitochondria and thus induces apoptosis of cancer cells. 5-FU, 5-fluorouracil; CTSG, cathepsin G; IL-8, interleukin 8; NET, neutrophil extracellular trap; PIK3CA, phosphatidylinositol-4,5-bisphosphate 3-kinase catalytic subunit alpha; RAGE, receptor for advanced glycation end products.

Oncogenic mutations often reprogram cancer metabolism. Cancer cells are addicted to glutamine.^30^ Upon entry into the cell via transporters, glutamine is converted to an ammonium ion and glutamate by mitochondrial glutaminases encoded by two genes in mammals, kidney-type glutaminase (GLS1) and liver-type glutaminase (GLS2). Glutamate can then be converted to α-ketoglutarate (α-KG), which enters the tricarboxylic acid cycle to generate ATP through the production of nicotinamide adenine dinucleotide (NADH) and flavin adenine dinucleotide (FADH2), by glutamate dehydrogenase (GLUD) or non-ammonia-producing aminotransferases.^30^ We discovered that *PIK3CA* mutations reprogram glutamine metabolism by up-regulating glutamate pyruvate transaminase 2 (GPT2) in colorectal cancer (CRC) cells through an AKT-independent, PDK1-ribosomal s6 kinase 2 (RSK2)-activating transcription factor 4 (ATF4) signaling axis, making them more dependent on glutamine^31^ (Fig. 3). Following *in vivo* infusions of [^13^C_5_]-glutamine in mice bearing subcutaneous colon cancer xenografts, we further verified that labeling from glutamine to most tricarboxylic acid cycle intermediates was higher in *PIK3CA*-mutant subcutaneous xenograft tumors than in wild-type *PIK3CA* tumors.^32^ We demonstrated that the glutaminase inhibitor CB-839 could preferentially inhibit xenograft growth of *PIK3CA*-mutant, but not wild-type, CRC.^33^ Moreover, the combination of CB-839 and 5-fluorouracil (5-FU) induces *PIK3CA*-mutant tumor regression in xenograft models.^33^ These data prompted phase I and II clinical trials of a combination of CB-839 and capecitabine, an oral prodrug of 5-FU. The phase I study shows that the drug combination is well-tolerated.^33^ Although not statistically significant due to the small sample size, patients whose tumors with a *PIK3CA* mutation show a trend of longer progression-free survival.^33^ The phase II clinical trial focused on *PIK3CA* mutant CRC patients. A subset of patients showed longer-than-expected progression-free survival.^34^ Mechanistically, CB-839 augments the anti-tumor effect by both the tumor's intrinsic and extrinsic pathways. Within the tumor cells, CB-839 treatment up-regulates uridine phosphorylation 1 (UPP1), which facilitates the conversion of 5-FU to fluorodeoxyuridylate (FdUMP), thereby inhibiting deoxythymidine triphosphate (dTTP) synthesis. Interestingly, the combination of CB-839 and 5-FU induces tumor regression in nude mice, but not in NOD scid gamma (NSG) mice,^34^ suggesting that innate immune cells modulate the anti-tumor effect of the drug combination. However, neither the depletion of natural killer cells nor macrophages impact the therapeutic effect of the drug combination. Surprisingly, the drug combination induces a large number of neutrophil extracellular traps (NETs), extracellular web-like structures of cytosolic and granule proteins assembled on de-condensed chromatin, in tumors in nude mice, but not tumors in NSG mice.^34^ NETs, formed by a process called Netosis, trap and kill bacteria, fungi, viruses, and parasites.^35^ We demonstrated that NETs play a key role in modulating the anti-tumor effect of the combination of CB-839 and 5-FU because disruption of NETs by either DNase I treatment or depletion of neutrophils in CRC tumors attenuated the efficacy of the drug combination. Mechanistically, the drug combination induced the expression of interleukin 8 (IL-8) preferentially in *PIK3CA* mutant CRC to recruit neutrophils into the tumors (Fig. 4). Moreover, the drug combination increased the levels of reactive oxygen species in neutrophils, thereby inducing NETs. Cathepsin G, a serine protease localized in NETs, enters CRC cells by binding to a cell surface protein, receptor for advanced glycation end products (RAGE). The internalized cathepsin G cleaves 14-3-3ε proteins, releases Bax, and triggers apoptosis in CRC cells.^34^ Furthermore, increased levels of NETs were associated with longer progression-free survival of the patients, suggesting that NETs induced by the drug combination inhibit tumor growth in patients.^34^

The physiological function of the IR-PI3K-AKT pathway is regulating blood glucose levels, and *PIK3CA* mutations activate the PI3K pathway and fuel cancer by promoting glucose uptake and accelerating glycolytic flux (Fig. 3). PI3K-AKT pathway promotes glucose uptake through various transporters, primarily glucose transporter 1 (GLUT1) and GLUT4.^36^ While GLUT1 is ubiquitously expressed, GLUT4 expression is mainly limited to insulin-responsive muscle and adipose tissue.^37^ So, the activated PI3K-AKT pathway enhances glucose uptake in cancer cells predominantly through GLUT1. On the one hand, the PI3K-AKT pathway regulates GLUT1 transcription through hypoxia-inducible factor 1α (HIF1α). On the other hand, the PI3K-AKT pathway phosphorylates and inactivates thioredoxin-interacting protein (TXNIP), which promotes endocytosis of GLUT1 and inhibits glucose uptake.^38^ Following uptake, hexokinase 2 (HK2) phosphorylates glucose, converting it into glucose-6-phosphate. This compound remains within cells and is poised to enter glycolysis. While the precise mechanism is not fully elucidated, the activated PI3K pathway enhances HK2 activity. Additionally, the PI3K pathway stimulates phosphofructokinase 1 (PFK1), the initial committed step in glycolysis, by phosphorylating and activating 6-phosphofructo-2-kinase/fructose-2,6-biphosphatase (PFKFB2). This activation results in the production of a potent allosteric activator of PFK1.^39^

## *PIK3CA* mutations alter the tumor microenvironment

**Figure 5:**
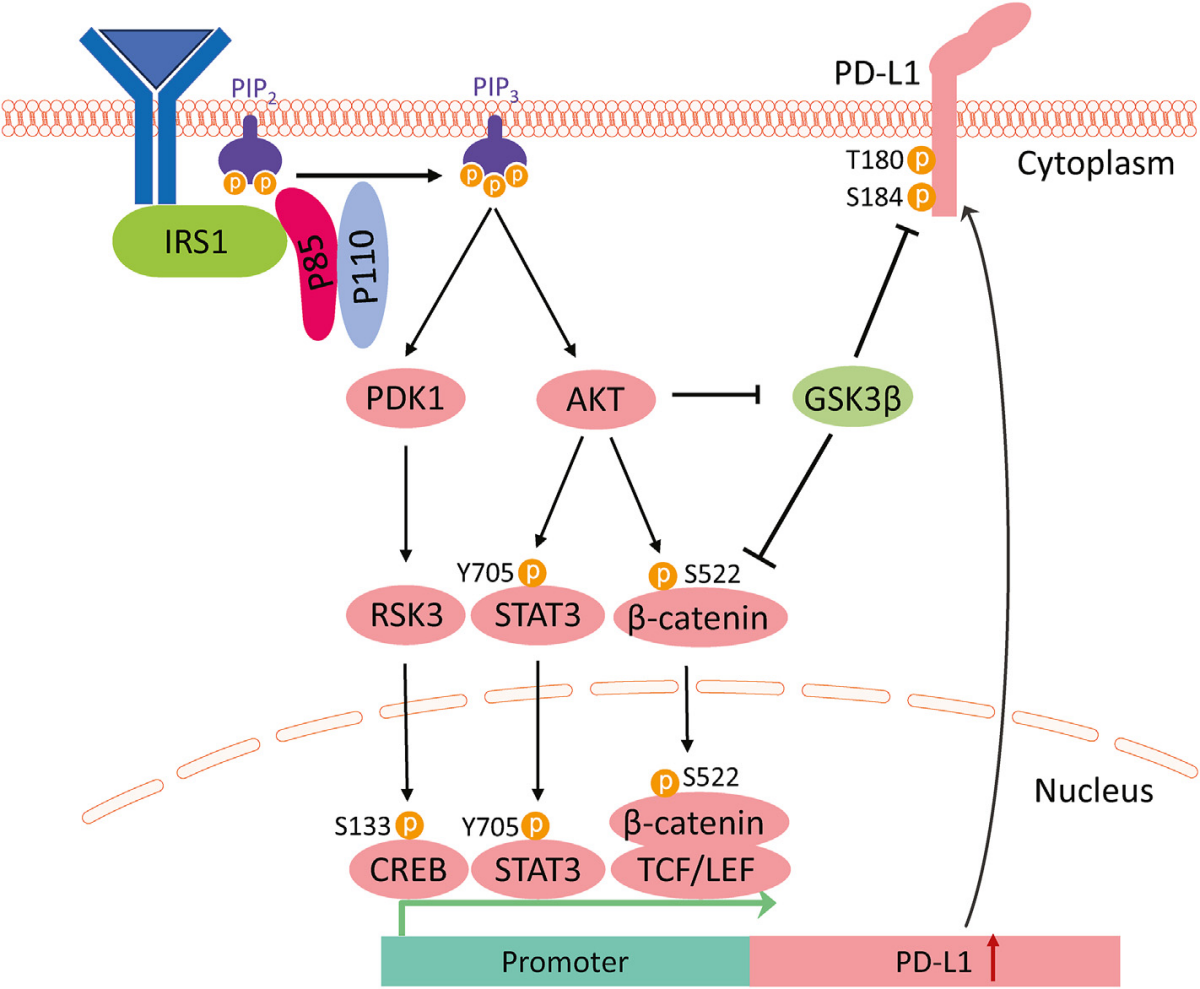
*PIK3CA* mutations alter the tumor microenvironment. PI3K pathway regulates PD-L1 expression through both PDK1 and AKT downstream pathways. ERBB3 activates PI3K through IRS1 and increases PDK1 activity to phosphorylate RSK3. Activated RSK3 then phosphorylates CREB at S133 and enhances transcription of PD-L1. Activated AKT phosphorylates STAT3 at Y705 and enhances its DNA binding affinity but not nucleus translocation. Activated AKT phosphorylates β-catenin at S522 and promotes its nucleus transportation and interaction with TCF/LEF. Both STAT3 and β-catenin contribute to the transcription of PD-L1. AKT regulates PD-L1 expression and stabilization by inhibiting GSK3β. On the one hand, AKT phosphorylates GSK3β and relieves the inhibition effect on β-catenin expression. On the other hand, AKT protects PD-L1 from phosphorylating by GSK3β and stabilizes PD-L1 from degradation. AKT, protein kinase B; CREB, cAMP response element-binding protein; ERBB3, Erb-B2 receptor tyrosine-protein kinase; GSK3β, glycogen synthase kinase 3β; IRS1, insulin receptor substrate 1; LEF, lymphoid enhancer-binding factor; PD-L1, programmed death-ligand 1; PDK1, phosphoinositide-dependent kinase 1; PI3K, phosphoinositide 3-kinase; PIK3CA, phosphatidylinositol-4,5-bisphosphate 3-kinase catalytic subunit alpha; PIP2, phosphatidylinositol (4,5)-bisphosphate; PIP3, phosphatidylinositol (3,4,5)-trisphosphate; RSK3, p90 ribosomal protein S6 kinase 3; STAT3, signal transducer and activator of transcription 3; TCF, T cell-specific factor.

Immunotherapy has become a very prospective treatment for cancer patients. However, the positive response only occurs in a fraction of patients and relies on dynamic interactions between tumor cells and immunomodulators inside the tumor microenvironment.^40^^,^^41^ The role of PI3Ks in proliferation, differentiation, and activity of immune cells has been well illustrated in recent reviews.^42^, ^43^, ^44^ We focus on how *PIK3CA* mutations in cancer cells help to remodel an immunosuppressive tumor microenvironment. Firstly, *PIK3CA* mutations arm cancer cells with the immune checkpoint regulator programmed death-ligand 1 (PD-L1), which transmits an inhibitory signal and reduces the proliferation of antigen-specific CD8^+^ T cells through binding to programmed cell death protein 1 (PD-1). Erb-B2 receptor tyrosine-protein kinase (ERBB3) mediates interferon-gamma (IFN-γ) induced PD-L1 expression through IRS1-PI3K-PDK1-p90 ribosomal protein S6 kinase 3 (RSK3)-cAMP response element-binding protein (CREB) signaling axis^45^ (Fig. 5). AKT-mediated phosphorylation of β-catenin on Ser522 promotes its nuclear translocation,^46^ where it interacts with T cell-specific factor (TCF)/lymphoid enhancer-binding factor (LEF) and activates PD-L1 expression.^47^ Meanwhile, AKT phosphorylates and inhibits GSK3β, which inhibits the transcriptional activity of β-catenin and enhances the degradation of PD-L1 by phosphorylating Thr180/Ser184 of PD-L1.^48^ Moreover, PTEN loss induced expression of immunosuppressive IL6, IL10, and PD-L1 in a PI3K-signal transducer and activator of transcription 3 (STAT3) axis dependent manner.^49^ Secondly, *PIK3CA* mutation in cancer cells creates an immunosuppressive stromal environment by induction of high glycolysis, leading to rapid consumption of glucose and the production of lactate. As a result, the depletion of metabolic fuels in the stroma and acidic environment contributes to immune suppression.^50^ On the one hand, high concentrations of lactate promote the expression of PD-1 in regulatory T cells, which could cause the failure of PD-1 blockade and tumor resistance.^51^ On the other hand, extracellular sodium lactate and lactic acid inhibit the motility of CD4^+^ and CD8^+^ T cells, respectively.^52^ In addition to metabolism, cancer cells harboring *PIK3CA* mutations domesticate immune cells directly through secreting growth factors, cytokines, and chemokines. The expression of vascular endothelial growth factor (VEGF) is tightly regulated by PI3K/mTOR,^53^ which not only promotes tumor vascularization but also enhances the infiltration of regulatory immune cells, including myeloid-derived suppressor cells (MDSCs),^54^^,^^55^ tumor-associated macrophages (TAM),^56^^,^^57^ and regulatory T cells.^58^ Moreover, VEGF directly reduces effector T cell activity,^59^^,^^60^ enhances regulatory T cell proliferation,^58^^,^^61^ and inhibits the maturing of dendritic cells.^62^^,^^63^

## Targeting *PIK3CA* mutant tumors with p110α isoform-specific inhibitors

Mutations in the *PIK3CA* gene are present in 28%–46% of people with HR^+^ HER2^−^ advanced breast cancer.^64^, ^65^, ^66^, ^67^ Thus, the combined treatment of a p110α-specific inhibitor alpelisib, and fulvestrant, an antiestrogenic medication, was tested in the SOLAR-1 phase III clinical trial. Although overall survival results did not meet the prespecified efficacy boundary in the SOLAR-1 trial, combined alpelisib and fulvestrant treatment statistically significant prolonged progression-free survival compared with placebo plus fulvestrant treatment (progression-free survival, 22.8 months *vs*. 18.2 months).^64^ Alpelisib is the only FDA-approved p110α isoform-specific inhibitor, which is in combination with fulvestrant to treat *PIK3CA*-mutated HR^+^ HER2^−^ breast cancer up to now.^3^ Alpelisib was also tested as monotherapy in heavily pretreated ER ^+^ HER2^−^ breast cancer and triple-negative breast cancer with *PIK3CA* mutations in a phase II clinical trial. The overall response rate was 30% in the ER^+^ HER2^−^ cohort, and 0% in the triple-negative breast cancer cohort.^68^ Decreasing mutant *PIK3CA* circulating tumor DNA (ctDNA) during treatment and estrogen receptor 1 (ESR1) mutations at baseline were related to a better prognosis, making them candidate biomarkers. However, side effects, including on-target side effects, restricted the maximum dose, and thus the clinical efficacy of alpelisib. In a managed access program (NCT0376573), 5 out of 8 patients had hyperglycemia (1 with grade 3) with fasting glucose levels of up to 450 mg/dL, 2 experienced rash (grade 1 and 3), and 2 with grade 3 diarrhea.^69^ Another study analyzed 27 patients treated with alpelisib between May 2019 and January 2022 and found that 24 out of 27 patients experienced adverse events, including 13 grade 3 adverse events (7 with hyperglycemia, 4 with rash, and 2 with diarrheas).^70^ Taselisib (GDC-0032) and inavolisib (GDC-0077) are two other p110α isoform-specific inhibitors in phase III clinical trials. A recent phase III study of inavolisib (INAVO120, NCT04191499) showed that inavolisib in combination with palbociclib, a cyclin-dependent kinase 4/6 (CDK4/6) inhibitor, and fulvestrant more than doubled progression-free survival compared with palbociclib and fulvestrant alone (15.0 months vs 7.3 months) in endocrine therapy-resistant *PIK3CA*-mutated HR^+^ HER2^−^ advanced breast cancer patients.^71^ Song et al profiled a selection of PI3K inhibitors for biochemical activity and pharmacokinetic properties and discovered that taselisib and inavolisib showed increased mutant potency in cell viability assays in a cancer cell line panel compared with other PI3K inhibitors.^72^ They further illuminated a unique mechanism of action of taselisib and inavolisib treatment leading to depletion of E545K/H1047R-mutant p110α protein but not wild-type (WT) p110α protein through HER2-p85β dependent ubiquitination and proteasome-mediated degradation.^72^ Although the underlying structural basis is still not clear and potency increased only 3-fold in *PIK3CA*-mutant isogenic cells versus parental WT cells, this discovery revealed a new mechanism of action to exploit in *PIK3CA*-mutant tumors. Other PI3Kα-specific inhibitors, including serabelisib^73^, ^74^, ^75^ and CYH33,^76^^,^^77^ are in the early stage of clinical trials.

## Targeting *PIK3CA* mutant tumors with mutant p110α-specific inhibitors

Although combined alpelisib and fulvestrant therapy has been approved by the FDA for the treatment of *PIK3CA*-mutated HR^+^ HER2^−^ breast cancer, side effects, including on-target side effects such as hyperglycemia, restricted the maximum dose and thus clinical efficacy of alpelisib. The percentages of patients who discontinued treatment owing to adverse events were 25% in the SOLAR-1 trial and 21% in the BYLieve trial.^78^^,^^79^ Thus, discovering PI3Kα mutant-specific inhibitors becomes a new direction of PI3Kα inhibitor development for the following reasons: E542/545K in the helical domain and H1047R in the kinase domain are two dominant hotspot mutations of *PIK3CA* gene accounting for over 50% mutations of *PIK3CA*; specifically targeting mutant PI3Kα can circumvent on-target side effects and may increase the clinical dose and thus efficacy.

RLY-2608 is the first allosteric pan-mutant-selective PI3Kα inhibitor that entered clinical trials. Andreas et al found a mutant-selective cryptic allosteric pocket of PI3K by molecular dynamics and cryo-electron microscopy. Then DNA-encoded libraries were applied to screen for mutant-selective inhibitors with orthosteric sites blocked by an inhibitor, and RLY-2608 was developed based on the hit found by the DNA-encoded libraries screening.^28^ In biochemical assays, RLY-2608 inhibits both kinase domain (H1047R) and helical domain (E542K and E545K) mutant PI3Kα activity with less than 10 nM potency and 8-to-12 times selectivity relative to WT PI3Kα. RLY-2608 is more than 1000-fold selective over the β, δ, and γ PI3K isoforms in biochemical assays and demonstrates exquisite selectivity across a panel of 322 kinases, with no other kinases showing over 50% inhibition.^28^ Notably, RLY-2608 at 100 mg/kg displayed anti-tumor activity similar to 50 mg/kg alpelisib but with a nominal effect on insulin and C-peptide levels in the mice models.^28^ Two patients from the ongoing dose-escalation portion of the first-in-human study of RLY-2608 (NCT05216432) showed partial response after receiving RLY-2608 for eight weeks without hyperglycemia related to WT PI3Kα inhibition.^28^ In particular, RLY-2608 could overcome resistance mediated by secondary mutations in *PIK3CA* identified in clinical patients who developed acquired resistance to PI3Kα inhibitors.^80^ These patient cases provide proof of concept that RLY-2608 is a first-in-class isoform- and mutant-selective allosteric inhibitor of PI3Kα.

STX-478, formerly ST-814, is an allosteric kinase domain mutant-selective PI3Kα inhibitor.^81^ Buckbinder et al found that STX-478 interacted with a previously undescribed allosteric pocket that formed due to a major conformational shift in residues 936–940, along with other smaller, local rearrangements.^82^ Although a detailed analysis of the allosteric site and key STX-478 interactions showed no difference between the WT- and H1047R-bound structures, the allosteric site occupied by STX-478 is more accessible in H1047R mutant as a surface plasmon resonance assay revealed that STX-478 had lower nanomolar binding affinity and faster association constants for H1047R mutant compared with E545K mutant and WT PI3Kα. STX-478 displayed about 15-fold selectivity for PI3Kα^H1047R^ versus PI3Kα^wt^ and 10-fold selectivity for PI3Kα^H1047R^ versus PI3Kα^E545K^ in binding affinity.^82^ Notably, STX-478 at 100 mg/kg displayed anti-tumor activity similar to 50 mg/kg alpelisib but with minimal effect on insulin-mediated glucose clearance in mouse model.^82^ A clinical study to evaluate the safety, tolerability, pharmacokinetics, and preliminary anti-tumor activity of STX-478 in participants with advanced solid tumors is recruiting and the results are yet to be identified (NCT05768139).

LOXO-783 is a potent, highly mutant-selective, and brain-penetrant allosteric PI3Kα H1047R inhibitor. LOXO-783 is exquisitely selective for PI3Kα H1047R, without inhibition on wild-type PI3Kα, other PI3K isoforms, or other kinases. No inhibition of phosphorylated AKT in WT cells was detected, even at high LOXO-783 concentrations. LOXO-783 has single-digit nanomolar (<5 nM) potency inhibiting proliferation of H1047R breast cancer cell lines, markedly higher cellular potency than alpelisib (150–320 nM). LOXO-783 causes significant tumor regressions in several PI3Kα H1047R-mutant ER^+^ HER2^−^ breast cancer models without causing significant weight loss or any increase in insulin or C-peptide.^83^ LOXO-783 shows promising additive effects for anti-tumor efficacy when combined with standard of care in breast cancers harboring the PI3Kα H1047R mutation. LOXO-783 is efficacious in ESR1-mutant as well as in abemaciclib and abemaciclib/fulvestrant double-resistant models.^84^ PIKASSO-01 is an ongoing phase I trial of LOXO-783 alone or in combination with anti-cancer therapies (NCT05307705).^85^

## Prospective

The PI3K pathway has fundamental roles in regulating proliferation, cell cycle, survival, metabolism, and motility.^1^ *PIK3CA* mutations remodel tumorigenic environments and promote tumor initiation and development. Activated PI3K pathway reprograms metabolisms of glucose and glutamine, two main fuels of cancer cells, and rebuilds an immunosuppressive tumor microenvironment. As *PIK3CA* is the most frequently mutated oncogene in cancer and is fundamental for cancer development, researchers show prompting interest in targeting the PI3Kα for cancer therapy. PI3Kα specific inhibitors have been developed rapidly in recent decades, and alpelisib has been approved by the FDA to treat HR^+^ HER2^−^ *PIK3CA*-mutated breast cancer in combination with fulvestrant. Nevertheless, on-target side effects pose limitations to its clinical efficacy, necessitating the exploration of alternative strategies. The identified hotspot mutations (E542/545K and H1047R) in *PIK3CA*, accounting for a significant portion of mutations, highlight the importance of developing mutant-specific inhibitors. Allosteric inhibitors that specifically target mutant PI3Kα developed fast recently, with three inhibitors tested in clinical trials, showing the promise of mutant-PI3Kα specific inhibitors. Targeting mutant PI3Kα-addicted pathway also circumvented the on-target side effects resulting from directly targeting PI3Kα kinase. In addition to allosteric mutant-specific inhibitors, CRISPR/Cas9-based gene editing is also a promising strategy to target the mutant *PIK3CA* gene at the genomic DNA level. CRISPR/Cas9 gene therapy targeting BCL11A (BCL11 transcription factor A) has been approved to treat sickle cell disease,^86^ which encouraged CRISPR/Cas9 therapies for other diseases resulting from specific gene mutations like *PIK3CA*. Yet, the formidable challenges lie in the delivery technology and *in vivo* application of CRISPR/Cas9 gene editing when it comes to treating solid tumors. In addition, small interfering RNA and antisense oligonucleotide therapies targeting mRNA are potential strategies if overcoming the hurdles of distinguishing a mutation in a single pair of nucleotides and mitigating potential off-target effects. These considerations not only contribute to a deeper comprehension of PI3Kα mutant-specific inhibitors but also open avenues for potential breakthroughs in cancer therapeutics.

## Funding

This work was supported by the 10.13039/100000002National Institutes of Health (USA) grants R01CA196643, R01CA264320, R01CA260629, P50CA150964, and P30CA043703 to Zhenghe Wang.

## Author contributions

**Yuxiang Wang:** Writing – original draft, Conceptualization. **Valery Rozen:** Conceptualization, Writing – original draft. **Yiqing Zhao:** Writing – original draft. **Zhenghe Wang:** Conceptualization, Writing – review & editing, Funding acquisition, Supervision.

## Conflicts of interests

Zhenghe Wang is the member of Genes & Diseases Editorial Board. To minimize bias, he was excluded from all editorial decision-making related to the acceptance of this article for publication. The remaining authors declare no conflict of interests.
